# The microbial biogeography of the gastrointestinal tract of preterm and term lambs

**DOI:** 10.1038/s41598-020-66056-z

**Published:** 2020-06-04

**Authors:** Clara Yieh Lin Chong, Tommi Vatanen, Mark Oliver, Frank H. Bloomfield, Justin M. O’Sullivan

**Affiliations:** 1 Liggins Institute, The University of Auckland, Auckland, New Zealand; 2 The Broad Institute of MIT and Harvard, Cambridge, MA USA; 3 Newborn Services, Auckland City Hospital, Auckland, New Zealand; 4 The Maurice Wilkins Centre, The University of Auckland, Auckland, New Zealand

**Keywords:** Health care, Paediatrics, Preterm birth

## Abstract

Nutritional supplementation is a common clinical intervention to support the growth of preterm infants. There is little information on how nutritional supplementation interacts with the developing microbiome of the small intestine, the major site for nutrient metabolism and absorption. We investigated the effect of preterm birth and nutritional supplementation on the mucosal and luminal microbiota along the gastrointestinal tract (GIT) of preterm lambs. Preterm lambs (*n* = 24) were enterally supplemented with branched-chain amino acids (BCAAs), carbohydrate (maltodextrin), or water for two weeks from birth. Term lambs (*n* = 7) received water. Mucosal scrapings and luminal samples were collected from the duodenum, jejunum, ileum (small intestine) and colon at six weeks post-term age and analysed by 16S rRNA amplicon sequencing. Anatomical site explained 54% (q = 0.0004) of the variance and differences between the term and preterm groups explained 5.7% (q = 0.024) of the variance in microbial beta-diversities. The colon was enriched with Tenericutes and Verrucomicrobia compared to the small intestine, while Actinobacteria, and superphylum Patescibacteria were present in higher abundance in the small intestine compared to the colon. Our findings highlight that early-life short-term nutritional supplementation in preterm lambs does not alter the microbial community residing in the small intestine and colon.

## Introduction

Successful GIT colonisation by specific bacterial populations depends on various factors including: host (genome and physiology of animals); bacterial (*e.g*. secretion of bacteriocins to eliminate other microorganisms), and environmental origin (*e.g*. diet)^1^. Diet has a dominant effect on the rumen microbial composition in cattle^2–4^. Studies in lambs have shown that early supplementation with different essential oils commencing at birth modulates the rumen microbial population measured post-weaning^5,6^. The microbial composition of the forestomach of ruminants has been well studied^7^; in contrast, the small intestine is rarely explored^8^. This is largely because international collaborations (*e.g*. Hungate1000, and Global Rumen Census) have focused on the rumen microbiome population in the effort to reduce methane emissions without affecting the economic baseline^3,9^.

Nutrition in early-life has been shown to play an essential role in shaping the gut microbiota^10^. For example, branched-chain amino acids (*i.e*. valine, isoleucine, and leucine) are essential dietary amino acids that are crucial for protein and neurotransmitter synthesis^11^. Nutritional supplementation with isoleucine has been shown to up-regulate the expression of the sodium glucose co-transporter (SGLT-1) in the duodenum, jejunum and ileum, and glucose transporter 2 (GLUT2) in the duodenum and jejunum of pigs^12^. In mice, a BCAA-enriched supplement alters the fecal abundance of health-promoting *Akkermansia* and *Bifidobacterium* and reduces the abundance of pathogenic bacteria such as *Enterobacteriaceae*^13^. However, the effect of BCAA supplementation on the gut microbiota of preterm mammals remains to be elucidated.

Unlike monogastric animals, sheep have a complex stomach comprising the rumen (first bioreactor), reticulum, omasum, and abomasum. The intestines are similar in structure to that of monogastric animals with a small intestine, caecum, colon, and rectum (see Supplementary Fig. S1). The rumen and reticulum form one functional unit, commonly known as the reticulorumen, due to the absence of a sphincter between the two^14^. The caecum and colon serve as another bioreactor with high bacterial activity for fermentation of cellulose material that escapes initial fermentation in the reticulorumen chamber^15^.

Despite differences in lamb and human physiology, preterm lambs offer a useful model system for understanding preterm birth in humans. In preterm lambs, nutritional supplementation alters early growth^16^. On the other hand, preterm birth alters pancreatic development and this change persists until adulthood^17^. However, a link between nutritional supplementation, microbiome and pancreatic development is missing. In humans, preterm birth, even at late preterm gestations, has life-long consequences with increased mortality from infancy to mid-adulthood^18^ and increased risk of adult-onset medical issues such as hypertension, diabetes, obesity, and cardiovascular-related problems^19^. Preterm babies are born with immature organs, lack nutritional stores (*e.g*. hepatic glycogen) and the commencement of breastfeeding is often delayed. Therefore, post-natal nutritional support is usually required to maintain energy and nitrogen balance, to support growth and to improve neurodevelopmental outcomes^20^.

Here, we used preterm and term lambs as a model system to investigate the effect of branched-chain amino acid and carbohydrate supplementation for the first two weeks after birth on the microbial community. We also characterised the biogeography of the mucosal and luminal microbiota from the duodenum, jejunum, ileum, and colon of preterm and term lambs at six weeks post-term age.

## Results

In total, we collected 228 samples from 31 lambs, 118 mucosal and 110 luminal samples. However, 140 samples had fewer than 3,000 sequencing reads after data cleaning and were excluded from further downstream analysis. Of the 88 samples that remained, 25 (28.4%; 21 colon, and 4 ileum) were mucosal and 63 (71.6%; 28 colon, 21 ileum, 6 duodenum, and 8 jejunum) were luminal samples.

### Anatomical sites drive differences in microbial community

**Figure 1:**
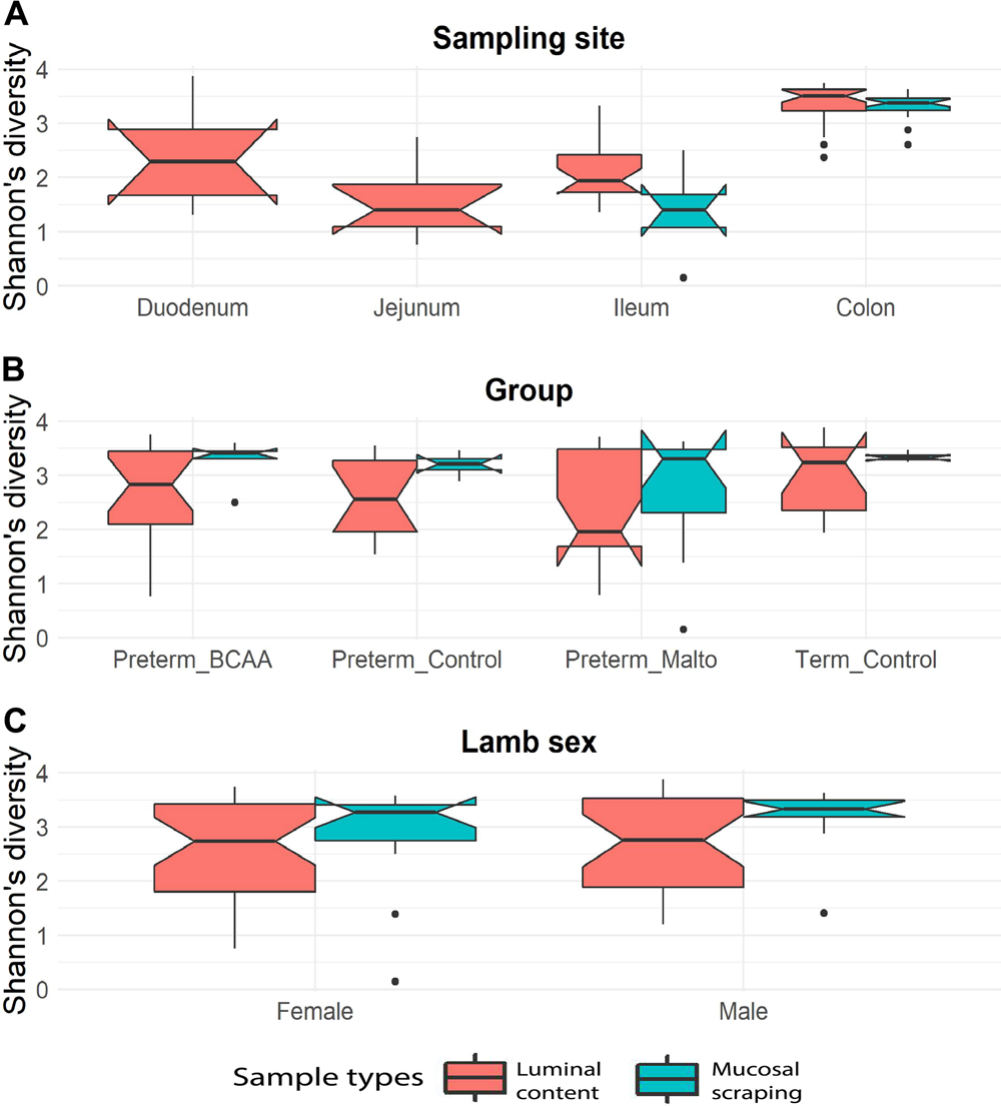
Shannon’s diversity index for mucosal scrapings (blue) and luminal content (red) at different anatomical regions of the gastrointestinal tract (**A**), amongst treatment group (**B**), and between sexes (**C**). Boxes indicate the interquartile range (IQR), the notch region shows the 95% confidence interval for the median, and the whiskers extending from the boxes represent the distribution within 1.5*IQR, with points beyond this range shown as outliers. The figure was generated using R software version 3.6.1 (R Core Team (2019). R: A language and environment for statistical computing. R Foundation for Statistical Computing, Vienna, Austria. (https://www.R-project.org/).

Microbial alpha diversity is a measure quantifying overall community complexity and is known to be affected by gut physiology^21^. We observed increasing microbial diversity, measured by Shannon’s diversity index, from the jejunum through to colon in the luminal content, and from the ileum to the colon for mucosal samples (Fig. 1A). The observed diversity was greatest in the colon. Microbial diversity varied according to different anatomical sites both overall (p = 1.5 × 10^−11^, adjusted p-value (q-value) = 7.5 × 10^−11^, Kruskal-Wallis test) and within the luminal content samples alone (p = 3.5 × 10^−8^, q-value = 8.7 × 10^−8^, Kruskal-Wallis test). We further conducted independent two group analyses between pairs of anatomical sites: colon harboured significantly higher diversity compared to duodenum (p = 2.2 × 10^−2^, q-value = 0.042, Mann Whitney test), jejunum (p = 7.9 × 10^−7^, q-value = 2.4 × 10^−6^), and ileum (p = 4.8 × 10^−10^, q-value = 2.9 × 10^−9^), and microbial diversity in the ileum was significantly higher than in the jejunum (p = 2.8 × 10^−2^, q-value = 0.042).

Microbial diversity within the colon and ileum mucosal scrapings showed significantly different compositions (p = 0.0002, q-value = 0.0006, Mann Whitney test). We were unable to compare diversities from the duodenum and jejunum within the mucosal scraping because low sequence reads meant the majority of samples were excluded from analysis. Notably, alpha diversity did not significantly differ between treatment groups overall (p = 0.407, Kruskal-Wallis test), when measured separately within the luminal content (p = 0.353, Kruskal-Wallis test), and again within the mucosal scraping (p = 0.822, Kruskal-Wallis test) (Fig. 1B). There were no differences in alpha diversity between female and male lambs overall (p = 0.551, Mann-Whitney test) or within luminal (p = 0.897) and mucosal (p = 0.291) samples (Fig. 1C). Microbial species richness estimate (Chao1) found statistically significantly differences amongst anatomical sites (q = 1.2 × 10^−8^, Kruskal-wallis test) but not between treatment groups, sex or between luminal and mucosal samples. These observations are consistent with the existing data describing the biogeography of GIT in different host organisms^22,23^.

### Microbial beta-diversity varied in the luminal content between ileum and colon

In order to understand the differences in the microbial composition along the GIT, we compared microbial beta-diversities between anatomical sites, luminal and mucosal samples, treatment groups (*i.e*. BCAA, maltodextrin, preterm and term control), and sexes using PERMANOVA. Overall, anatomical site explained 54% (q = 0.0004) of the variance while term and preterm groups explained 5.7% of the variance (q = 0.024) in beta-diversities. There were no significant differences between luminal and mucosal samples (q = 0.215) or male and female lambs (q = 0.172).

**Figure 2:**
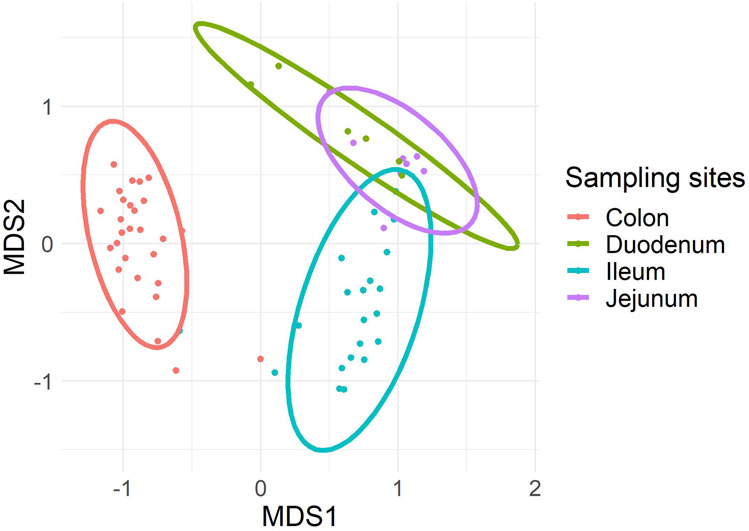
Principal coordinate analysis of β-diversity identified clustering of microbial profiles in luminal samples based on anatomical sites (q = 0.0003). *MDS: multidimensional scaling. The figure was generated using R software version 3.6.1 (R Core Team (2019). R: A language and environment for statistical computing. R Foundation for Statistical Computing, Vienna, Austria. (https://www.R-project.org/).

We further analysed the beta-diversity of the microbial populations from the mucosal scraping and luminal content separately. We found that the anatomical site explained 54% (q = 0.0003) of the variance in luminal content samples (Fig. 2). Sex of the lambs or different treatment groups did not contribute significantly to the variance in luminal content (R^2^ = 0.013, q = 0.129 and R^2^ = 0.039, q = 0.084, respectively). Because the majority of mucosal samples for both the duodenum and jejunum had low reads, we were unable to undertake comparisons of luminal and mucosal microbiomes at these sites.

The microbial diversity (Bray-Curtis) in the luminal content between ileum and colon was significantly different (R^2^ = 0.48, q = 0.0001). Similarly, microbial beta-diversity was significantly different between the ileum and either the jejunum (R^2^ = 0.326, q = 0.0002) or duodenum (R^2^ = 0.282, q = 0.0002). However, the beta-diversity in the jejunum and duodenum was not significantly different (R^2^ = 0.117, q = 0.178). Similarly, the microbial diversity (Bray-Curtis) between the mucosal scrapings and luminal content was not significantly different in either the ileum (R^2^ = 0.089, q = 0.13) or colon (R^2^ = 0.021, q = 0.37).

### Biogeographical delineation of microbial composition at different taxonomical levels

**Figure 3:**
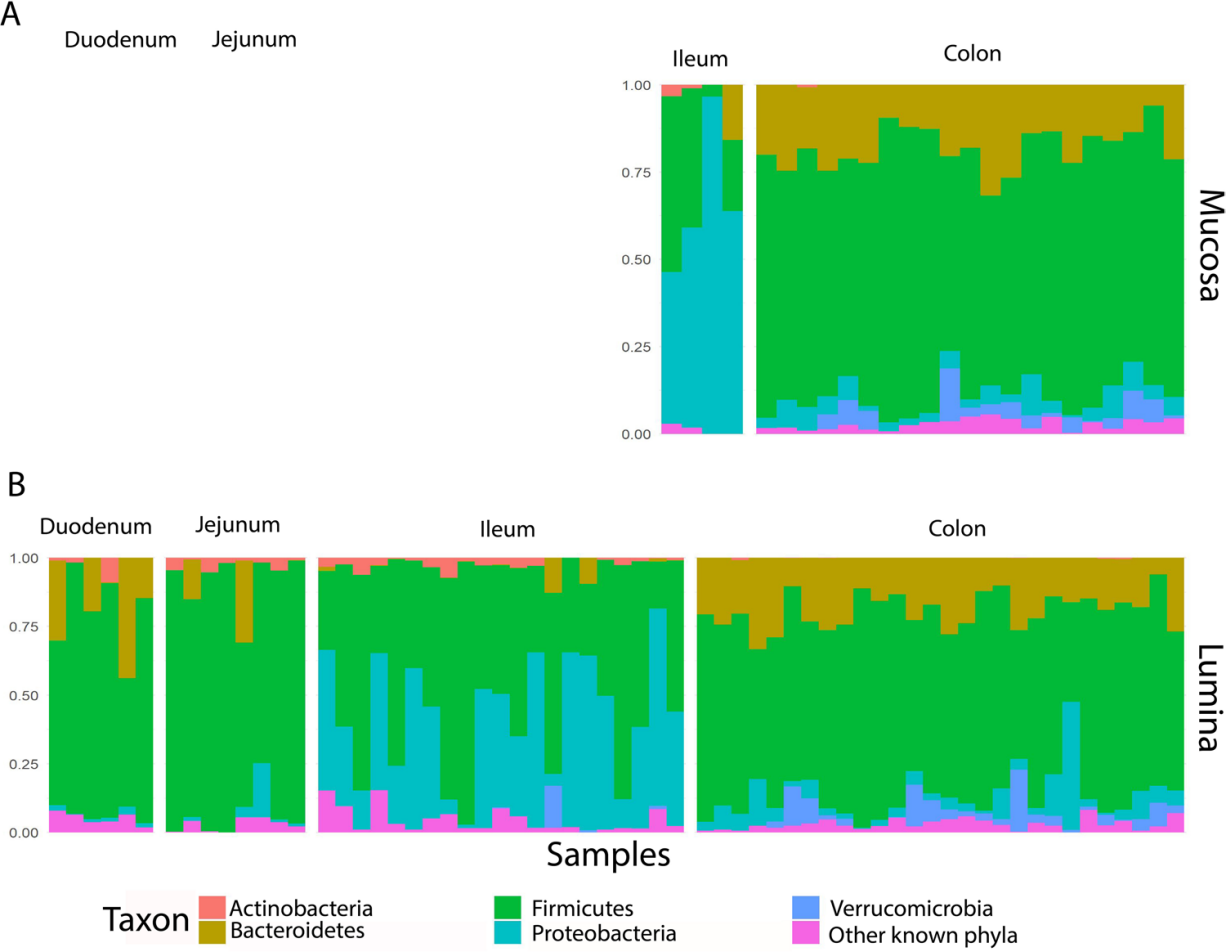
The five most abundant microbial phyla, with mean relative abundance >0.9%, grouped according to sample type (**A** mucosa; **B** lumina) across each anatomical site. Mucosal samples: ileum, *n* = 4; colon, *n* = 21. Lumi*n*al content samples: duodenum, *n* = 6; jejunum, *n* = 8; ileum, *n* = 21; colo*n*, *n* = 28. The figure was generated using R software version 3.5.0 (R Core Team (2018). R: A language and environment for statistical computing. R Foundation for Statistical Computing, Vienna, Austria. (https://www.R-project.org/).

Firmicutes was the most abundant phylum throughout the GIT from the small intestine to the colon (Fig. 3). Actinobacteria, and Patescibacteria were more abundant in the small intestine compared to colon. The colon was enriched with members of the Verrucomicrobia, and Tenericutes compared to the small intestine. Bacteroidetes, Tenericutes, Verrucomicrobia, Planctomycetes and Firmicutes were present at greater abundance in the colon than in the ileum. By contrast, Proteobacteria, Actinobacteria, Patescibacteria, Euryarchaeota (Archaea) and Cyanobacteria were more abundant in the ileum.

When mucosal scrapings and luminal samples were analysed separately, the genera *Lactobacillus, Acetitomaculum*, and *Lachnospiraceae NK3A20 group* from the phylum Firmicutes and genus *Candidatus Saccharimonas* from the phylum Patescibacteria were enriched throughout the luminal content in the small intestine (q value < 0.05) when compared with that of the colon. In the colon luminal content, four genera from the phylum Bacteroidetes*;* 22 genera from the phylum Firmicutes*;* one order from phylum Tenericutes, and one genus from the phylum Verrucomicrobia were present at high abundance compared to small intestine (duodenum, jejunum, and ileum) (q value < 0.05, see Supplementary Table S1 and Supplementary Fig. S2).

### The effect of preterm birth and early-life supplementation on the microbial composition

The effect of early-life supplementation was examined by taking into account the possible effect of preterm birth. Preterm birth was associated with microbial variation when compared with lambs born at term (R^2^ = 0.057, q = 0.024). When compared to lambs born preterm, lambs born at term had, on average, a greater abundance of genera within the phylum Firmicutes: *Lachnoclostridium, GCA_900066225, Ruminococcus gauvreauii group, Tyzzerella 4, Ruminococcus torques group*, and *Erysipelatoclostridium*. Among these six genera, four belong to the family *Lachnospiraceae* (*Ruminococcus gauvreauii group, Tyzzerella 4, Ruminococcus torques group*, and *Lachnoclostridium*), one from *Ruminococcaceae* (*GCA_900066225*), and one from *Erysipelotrichaceae* (*Erysipelatoclostridium*)*. Ruminococcus torques group*, and *GCA_900066225* were present in greater abundance in the colon of lambs that were born at term; *Tyzzerella 4*, and *Erysipelatoclostridium* were enriched in the mucosal scraping of term lambs; while *Lachnoclostridium* was present mostly in the colonic luminal content of term-born lambs (Supplementary Table 2).

Two weeks of nutritional supplementation of preterm lambs after birth did not alter the microbial communities when compared to the control preterm lambs that had water (BCAA versus control: q = 0.22; Malto versus control: q = 0.11). This is consistent with the effect of preterm birth predominating over the nutritional influence on the microbial communities in the GIT of the lambs.

## Discussion

An increase in alpha diversity of the microbial populations from the jejunum through to the colon of young lambs is consistent with previous studies of 30-day pre-weaned lambs^24^ and 12-month-old Small-tail Han sheep (n = 3)^25^. The small intestine is a chamber mainly for enzymatic reactions, digestion, and absorption^1^. The acidic digesta carried from the abomasum^15^ into the small intestine may create a hostile environment for microorganisms explaining the low observed diversity. The alkaline liquid secretions that enter the duodenum from the pancreas contain enzymes, while those that enter from the liver contain bile salts and organic substances^15^ that neutralise the acidic environment and contribute to the progressively increasing microbial diversity.

We did not observe a significant difference in terms of microbial composition between luminal and mucosal samples from the ileum. It is possible that the contraction that occurs along the length of the GIT, in order to move the disgesta, creates a mixing effect that explains the similarity in the microbial populations observed between mucosal and luminal samples in both the ileum and colon. However, it is difficult to extrapolate and predict the effects of this mixing at the microbial scale. Unfortunately, most of the mucosal samples from both the duodenum and jejunum had low sequencing reads and were excluded from downstream analysis. This was despite the quality and quantity of extracted DNA in our study falling within the required range for sequencing. We contend that the low number of sequencing reads generated indicates that non-bacterial DNA was also extracted. This problem can be resolved by: a) amplification of the full-length 16S rRNA gene followed by nested amplification of the V4 region to obtain a satisfactory amount of PCR products^24^; or b) shot-gun metagenomic sequencing of the samples to enable identification of the other DNA fraction; or c) using the methyl-CpG binding domain (MBD) to enrich the microbial DNA by separating it from the host DNA^26^.

We observed increased amounts of Cyanobacteria in the ileum compared to the colon. Cyanobacteria are ubiquitous environmental bacteria and ruminants ingest them while grazing on plants^27^. For example, in camels, Cyanobacteria were detected in eight different segments of GIT including the ileum^22^. Cyanobacteria are photosynthetic and it is unlikely that any Cyanobacterial cells survive the GIT environment. It is possible that these environmental origin Cyanobacterial cells and cell-free Cyanobacterial DNA are being degraded while passing through the GIT. We contend that this explains our observation of reduced Cyanobacterial abundance in the colon.

The small intestine harboured a high abundance of *Lactobacillus* compared to the colon, consistent with previous observations^28^. *Lactobacilli* are widely available in many milk products originating from animals and is a promising candidate for probiotic effects^29^. Digesta that enter the small intestine come from the abomasum, the true stomach similar to a monogastric animal. In newborn ruminants, suckling induces a reflex contraction of the reticular groove muscles, causing milk entering the oesophagus to bypass the reticulorumen and move directly into the abomasum^15^. Thus, the significantly high abundance of this genus in the small intestine compared to the colon may be explained by the milk-enriched diet of the lambs^24^.

Wang *et al*. (2016) reported that *Lachnospiraceae* and *Ruminoccocaceae* are among the most dominant families present in the colon of 10-month old sheep^30^. Notably, the majority of the observed genera that were present at significantly higher numbers in term-born compared to preterm lambs in our study belong to the family *Lachnospiraceae* and *Ruminoccocaceae*. It remains possible that prematurity, which includes the degree of gut maturation and time of weaning, contributes directly to this phenomenon. For example, the gut undergoes significant maturation in late gestation and preterm lambs are born with less mature intestinal tracts^31^. Weaning of lambs varies between 6 and 12 weeks^5,6,16^ and may be impacted upon by maturation of feeding behavior and of the intestine. The extent of the impact of preterm birth may have been ameliorated by induction with corticosteroids, as these are known to accelerate maturation of the foetus. However, all lambs were treated identically except for the different nutritional supplementation received in each group. Moreover, all ewe-lamb dyads were returned to pasture within a week of birth with consequent exposure to the pastural microbial flora. As lambs were on pasture, we do not have data on the age at which lambs began to ingest grass or weaned from their mothers. Therefore, as term lambs are theoretically more mature compared to preterm lambs, it remains possible that they ingested more grass and consequently, their microbiome composition more closely resembled that of older sheep.

In this study, we did not observe any significant difference in the microbial population between preterm lambs with and without supplementation in the small intestines and colon. Nutrient absorption in ruminants mainly takes place in the foregut which includes the rumen, reticulum, and omasum^15^. However, it is likely that most of the supplement these lambs received bypassed the rumen, reticulum, and omasum and instead passed directly to the abomasum and small intestine. This diversion is due to the reticulo-oesophageal groove reflex that is elicited by sucking behaviour but also by other conditional response stimuli associated with regimented feeding^32^. Minor latency in the reflex may allow some supplement to enter the reticulorumen, but very little is likely within the experimental paradigm employed in these studies. Crucially, the observation that the microbial population did not differ between the BCAA supplemented and non-supplemented groups in the small intestines and colon does not mean that the active microbial metabolic pathways were the same between groups. A more comprehensive study with metatranscriptomic and metagenomics would provide definitive evidence that the supplemental BCAAs were impacting on the microbial population. This could be supplemented by evidence for differential microbial fermentation in the form of circulating metabolic products (*e.g*. volatile fatty acids [acetic, propionic, and butyric]). Quantitative information by using flow cytometry or quantitative polymerase chain reaction (qPCR) to measure bacteria load^33^, could also act as an auxiliary data further improving the interpretation of 16S amplicon results.

## Conclusion

This study provides an insight into the effect of biogeographical differences and the effect of prematurity on the microbial population along the GIT of lambs at six weeks post-term age. We conclude that different anatomical sites, from small intestine to colon, harbour distinct microbial communities and that early-life short-term nutritional supplementation had no effect on the microbial populations of preterm lambs. Our study indicates that prematurity itself, a phenomenon where there is early exposure to the extra-uterine environment together with an under-developed gut physiology, contributes to the distinct microbial community in preterm infants. However, future studies should isolate the effects of medical interventions (*e.g*. antibiotics) and the hospital environment on preterm microbiome development.

## Materials and methods

### Ethics approval

This study was approved by the Animal Ethics Committee of the University of Auckland (AEC protocol number 001525). All experiments were conducted in accordance with our Institutional Code of Ethical Conduct which is based on the requirements of NZ Animal Welfare Act Legislation (1999, including subsequent amendments) and under the direction of the NZ Ministry of Primary Industries. The obligations under these regulations are compatible with those of ARRIVE (Animal Research: Reporting of *In Vivo* Experiments) and other internationally accepted standards.

### Experimental design and Sample collection

Five-year-old Romney singleton-bearing ewes of known gestation were acclimatised to concentrate feed and indoor housing. Ewes were randomly assigned to their experimental groups (term or preterm delivery) by ballot, where treatment group tags were placed in opaque envelopes, randomly sorted and drawn, once they had successfully acclimatised to the feedlot. Term gestation is typically 147 days. Labor was induced in all animals by intramuscular injection of dexamethasone phosphate (0.25 mg/kg per dose, PHENIX, KELA N.V.) on days 135 and 136 of pregnancy (preterm groups) and on days 145 and 146 (the term group) leading to birth at 137 ± 0.5 and 147 ± 0 days respectively.

Newborn preterm lambs were randomly assigned, using the ballot system mentioned above, straight after birth to one of three groups: preterm control, preterm BCAA, and preterm maltodextrin. The Preterm BCAA group (n = 9) received a 2:1:1 formulation of leucine:isoleucine:valine while the Preterm malto group (n = 10) received an isocaloric quantity of the carbohydrate maltodextrin, both mixed in approximately 20 mL water given in 2 aliquots per day for the first 14 days after birth. Preterm control (n = 5) and term control lambs (n = 7) received 20 mL water to ensure all lambs were handled identically and exposed to an equal volume of additional liquid. In addition to the supplements, all lambs suckled at their ewe *ad libitum*. Following the two week supplementation period, ewes and lambs were returned to outdoor pasture.

At six weeks of age, lambs were euthanised by lethal overdose of injectable anesthetic (Pentobarbitone 80 mg/kg, PROVET) and samples of the duodenum, jejunum, ileum, and colon were collected. The duodenum was identified as the part of the small intestine immediately distal to the common bile duct; the jejunum sample was taken from the small intestine 20 to 30 cm distal to the duodenum; the ileum from 30 cm proximal to the caecum, and colon from 30 cm distal to the caecum. The luminal content was collected from each anatomical site, transferred into a 2.0 mL cryovial tube (Thermo Fisher), and snap-frozen in liquid nitrogen. Mucosal scrapings were collected by opening out a 2 cm piece of intestine and gently scraping the internal wall with a sterile scalpel blade (#11). Mucosal scrapings were transferred into a cryovial tube and snap-frozen in liquid nitrogen. Once collected, all samples were transported to The Liggins Institute, The University of Auckland, New Zealand on dry ice.

### DNA extraction and 16S rRNA amplicon sequencing

DNA was extracted from 228 mucosal and luminal samples using the QIAamp PowerFecal DNA kit (QIAGEN) according to the manufacturer’s protocol. The quality and quantity of the extracted DNA were measured using a NanoPhotometer N60 (IMPLEN, Germany) and a Qubit (Invitrogen, US) prior to 16S rRNA amplicon library preparation.

16S rRNA amplicon libraries were prepared using the Nextera XT kit (Illumina). The V3-V4 hypervariable region was amplified using the universal primers 341 F (5′-CCTACGGGNGGCWGCAG-3′) and 805 R (5′-GACTACHVGGGTATCTAATCC-3′). 16S rRNA amplicon sequencing was performed on an Illumina MiSeq sequencing platform. Unprocessed sequences were deposited in the SRA under the Project Accession Number SUB6650612 or PRJNA594238.

### Bioinformatics and statistical analysis

16S rRNA amplicon sequencing data were processed using QIIME2 (v2019.4)^34,35^. Briefly, sequence quality control and denoising were performed using DADA2^36^, including removal of the PhiX reads and chimeric sequences. The resulting amplicon sequence variants (ASVs) were taxonomically annotated using the SILVA database (release 132_99). Samples that were included in downstream analyses had filtered sequence counts ranging from 3,007 to 30,267 reads. 140 samples had fewer than 3,000 sequencing reads and were excluded from further analysis.

Statistical analyses were conducted using R version 3.5.0. Permutational multivariate analysis of variance (PERMANOVA; adonis function from the vegan package in R, 10,000 permutations) was used to quantify the contribution of different covariates to the observed variance in microbial beta-diversities. Associations between individual microbial taxa that were present in more than 20% of the samples and extrinsic covariates (*i.e*. sampling sites, treatment groups, types of samples, and sexes of the lambs) were tested using Multivariate Association with Linear Models (MaAsLin)^37^. MaAsLin enables controlling for possible confounding factors; in this case, it is the repeated sampling from the same lambs. We used Mann Whitney and Kruskal-Wallis test for independent two groups and more than two groups comparison respectively. All q-values (fdr corrected p-values) reported in this manuscript were corrected for multiple testing using Benjamini-Hochberg procedure^38,39^.

## Supplementary information

Supplementary Table 1.

Supplementary Table 2.

Supplementary Figure 1.
