# Supplementary material for: The microbial biogeography of the gastrointestinal tract of preterm and term lambs
Source: Sci Rep. 2020 Jun 4;10:9113. doi: 10.1038/s41598-020-66056-z (PMC7272652; doi:10.1038/s41598-020-66056-z)
Supplement: Supplementary file 3 — Supplementary Figure 1. [file 41598_2020_66056_MOESM3_ESM.pdf]

Title: The microbial biogeography of the gastrointestinal tract of preterm and term lambs

Clara Yieh Lin Chong[1], Tommi Vatanen*[1,2], Mark Oliver[1], Frank H. Bloomfield[1,3], Justin M. O'Sullivan*[1,4]

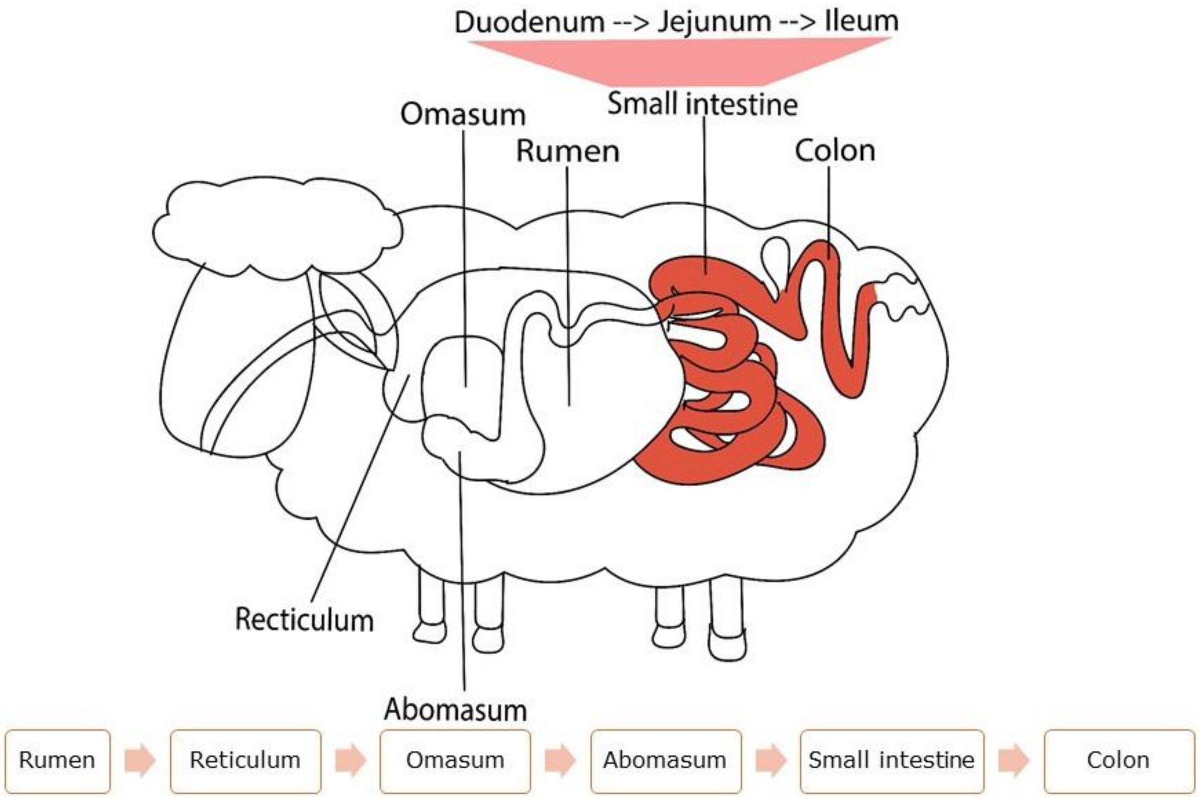

Supplementary Figure 1: Anatomical sites along the gastrointestinal tract of a lamb. The section illustrated in red was examined in this study. The flow of ingested feed is illustrated at the bottom of the figure.

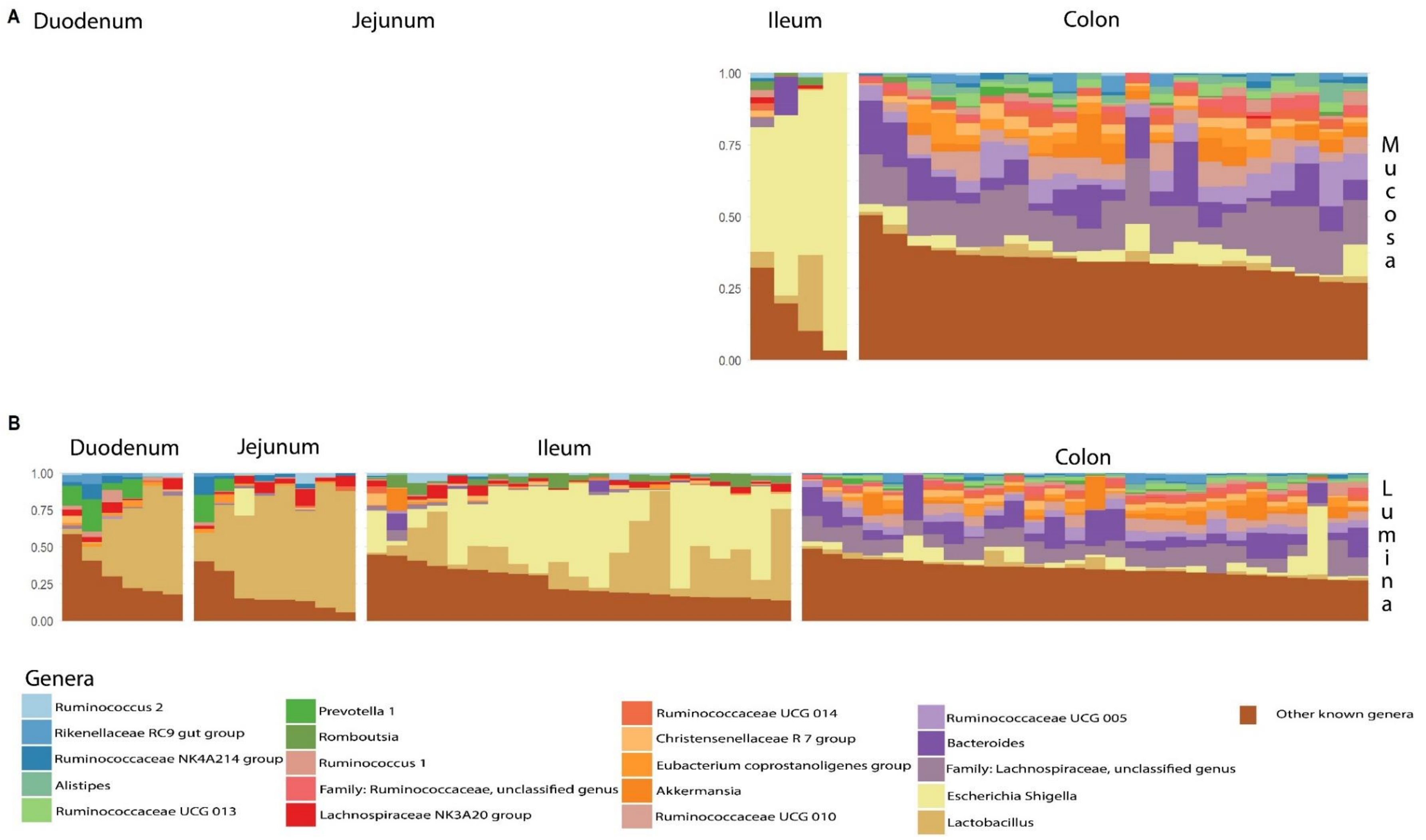

Supplementary Figure 2: The 20 most abundance microbial genera, with mean relative abundance >0.28%, grouped according to mucosal or luminal samples across each anatomical site. The figure was generated using R software version 3.6.1 (R Core Team (2019). R: A language and environment for statistical computing. R Foundation for Statistical Computing, Vienna, Austria. (https://www.R-project.org/).
